# Editorial: The role of calcium and calcium binding proteins in cell physiology and disease

**DOI:** 10.3389/fphys.2023.1228885

**Published:** 2023-06-09

**Authors:** N. Lowri Thomas, C. Dart, N. Helassa

**Affiliations:** ^1^ School of Pharmacy and Pharmaceutical Sciences, Cardiff University, Cardiff, United Kingdom; ^2^ Institute of Systems, Molecular and Integrative Biology, University of Liverpool, Liverpool, North West England, United Kingdom; ^3^ Department of Cardiovascular and Metabolic Medicine, Institute of Life Course and Medical Sciences, Faculty of Health and Life Sciences, University of Liverpool, Liverpool, North West England, United Kingdom

**Keywords:** ryanodine receptors (RyRs), store operated Ca^2+^ entry, mitosis, Ca^2+^ signalling, TRP channel, er stress, channelopathies

Ca^2+^ is possibly the most versatile and universal signalling agent in cell physiology. It controls several biological processes: it triggers life at fertilization, and controls the proliferation, development and differentiation of cells, as well as regulating diverse processes such as secretion, metabolism, muscle contraction, neuronal excitability, learning and memory, and cell death (Berridge et al., 2000). To coordinate all of these functions, Ca^2+^ signals need to be flexible yet precisely regulated in time and space. This is achieved by a variety of ion channels, pumps, transporters and Ca^2+^ binding proteins. This results in complex, dynamic signals that can be easily measured using chemical dyes (Grynkiewicz et al., 1985) when changes are of a global nature, but when they are more localised (i.e., Ca^2+^ signalling micro or even nanodomains (Bootman and Bultynck, 2020), they become more difficult to quantify by standard methods.

In their review, Nugues et al. discuss this in the context of Ca^2+^ signals that regulate the process of mitosis. Detection of these short-lived, spatially limited signals was only made possible due to the development of genetically encoded Ca^2+^ indicators. These probes capitalise on the Ca^2+^-binding properties of calmodulin (CaM), together with green-fluorescent protein-based fluorophores and can be targeted to specific organelles or cellular compartments to measure localised Ca^2+^ changes (Heim and Tsien, 1996; Miyawaki et al., 1997). The review describes how these reporters could be used to pinpoint Ca^2+^ sensitive processes in mitosis, such as when GCaMP6 tethered to actin, nucleated at centrosomes and detected Ca^2+^ signals thought to play a role in orienting the mitotic spindle (Farina et al., 2016; Helassa et al., 2019; Lagos-Cabre et al., 2020). The authors also emphasise the importance of Ca^2+^ binding proteins in mitosis, dysregulation of which may affect protein kinase activation (Lee et al., 2014; Zhou et al., 2019), altered gene expression and ultimately oncogenesis and the development of anti-cancer drugs which target Ca^2+^ signalling components is a burgeoning field of therapeutics (Roderick and Cook, 2008; Monteith et al., 2017).

The process of store-operated Ca^2+^ entry (SOCE), where depletion of intracellular Ca^2+^ stores—sensed and communicated by stromal interaction molecules (STIMs)—triggers Ca^2+^ influx via Orai channels, is a fundamental mechanism in maintaining cellular Ca^2+^ homeostasis. In this Research Topic, Manning et al. review the role of SOCE in skin physiology and pathophysiology. In the skin, SOCE regulates the processes of proliferation, differentiation, melanogenesis and sweat secretion (Vandenberghe et al., 2013; Stanisz et al., 2016; Evans et al., 2018) and changes to the molecular components of SOCE can lead to pathological outcomes, e.g., psoriasis, anhidrosis and potentially melanoma (Leuner et al., 2011; Hooper et al., 2015; Concepcion et al., 2016). In their brief research report Manning et al. describe the clustering of TRPC1 (Transient Receptor Potential Cation Channel Subfamily C Member 1) channels, which mediate the Ca^2+^ influx that drives the differentiation of keratinocytes (Fatherazi et al., 2007; Beck et al., 2008; Müller et al., 2008). Using immunogold transmission electron microscopy of keratinocyte plasma membrane sheets, they showed evidence that during store depletion/SOCE, TRPC and Orai1 subunits form separate clusters that move towards each other. The authors suggest that the grouping of TRPC channel subunits supports the theory that STIM interacts with TRPC1 to initiate this current, with the formation of Orai-TRPC-STIM complexes and the insertion of constitutively active TRPC channels being less likely mechanisms of activation.

The second brief research report in this Research Topic concerns the discovery that ryanodine receptor Ca^2+^ release channel dysfunction in a neuromuscular disorder [malignant hyperthermia (MH)] and a cardiac arrhythmia syndrome [catecholaminergic polymorphic ventricular tachycardia (CPVT)], result from similar molecular mechanisms. Using chemical cross-linking reactions, Zhang et al. demonstrated intra-subunit interactions within the tetrameric structure of the channel are disrupted as a result of mutation which causes amino acid substitution in the N-terminus of the protein [R163C in the skeletal muscle isoform, RyR1 (Quane et al., 1993) and R169Q in the cardiac isoform, RyR2 (Hsueh et al., 2006)]. This interaction is thought to be critical in maintaining the closed state of the channel, and when disrupted leads to a ‘Ca^2+^ leak’, culminating in a pathological outcome in both disorders (Tung et al., 2010; Zissimopoulos et al., 2013; Zissimopoulos et al., 2014). Moreover, they also showed that it was the positive charge of the substituted amino acid that was instrumental in maintaining that interaction, since the introduction of other positively charged amino acids at the subunit interface allowed domain tetramerization to occur as normal. This evidence lends credibility to the cryo-electron microscopy structures used to map the subunit interfaces (des Georges et al., 2016; Peng et al., 2016), and also suggests that the development of pharmacological tools that targets this interaction may be beneficial therapeutically.

RyR2 dysfunction is a known arrhythmia trigger and Hamilton and Terentyev discuss how endoplasmic reticulum (ER) stress can affect this phenomenon, as well as regulation of sarco (SR)/endoplasmic reticulum Ca^2+^ store homeostasis in general. It seems logical that, since the SR/ER is the site of protein processing, stress-responses in this organelle can have a profound and ultimately pro-arrhythmic effect on Ca^2+^ handling proteins and ion channels. The unfolded protein response (UPR) is a signal transduction system that upregulates various stress-response proteins (e.g., chaperones) in response to conditions (e.g., ischaemia, changes in SR Ca^2+^ levels) that impact the efficiency of protein folding in the rough ER (Glembotski, 2008). While in the short term this response increases protein folding capacity, chronic ER stress can mean that the UPR results in excessive production of reactive oxygen species (ROS), that can in turn modify SR/ER proteins, including RyR2 and the SR Ca^2+^ ATPase pump, thereby altering SR Ca^2+^ homeostasis (Chin et al., 2011). This can be achieved directly, via redox modification of cysteines (Lancel et al., 2009; Cooper et al., 2013; Hobai et al., 2013)—which in turn can also affect accessory protein mediated regulation of function (Nikolaienko et al., 2020; Hamilton et al., 2022), or by aberrant regulation via ROS-activated protein kinases (Liu et al., 2014; Hegyi et al., 2021). Given that such UPR-dependent changes have been shown to contribute to cardiomyopathy, heart failure and ischaemic injury as well as arrhythmogenesis (Glembotski, 2008; Liu et al., 2014; Wiersma et al., 2017; Liu et al., 2021), it will be essential to study further the relationship between Ca^2+^ handling and SR/ER stress proteins may be when developing new therapeutic approaches for cardiovascular disease.

The articles included in this Research Topic reinforce that Ca^2+^ signalling remains a growing area of research with still much scope for the development of both diagnostic and therapeutic tools that target its component parts.
